# Impact of Distracting Emotional Stimuli on the Characteristics of Movement Performance: A Kinematic Study

**DOI:** 10.3389/fpsyg.2021.642643

**Published:** 2021-03-25

**Authors:** Yingzhi Lu, Tianyi Wang, Qiuping Long, Zijian Cheng

**Affiliations:** ^1^School of Psychology, Shanghai University of Sport, Shanghai, China; ^2^Brain and Cognitive Neuroscience Research Center, Liaoning Normal University, Dalian, China

**Keywords:** continuous movement, discrete movement, accuracy, RMSE, emotion

## Abstract

It is well-documented that emotional stimuli impact both the cognitive and motor aspects of “goal-directed” behavior. However, how emotional distractors impact motor performance remains unclear. This study aimed to characterize how movement quality was impacted during emotional distractors. We used a modified oddball paradigm and documented the performance of pure movement. Participants were designated to draw a triangle or a polygon, while an emotional stimulus was presented. Speed was assessed using reaction time and movement time. The quality and precision of movement were assessed by calculating the accuracy and root-mean-square error (RMSE). Compared to drawings of triangles, polygons had higher accuracy under negative stimuli, but lower RMSE under positive stimuli. The results indicate that distracting emotional stimuli impact different aspects of movement quality, with movement complexity influencing accuracy under negative distractors and precision under positive distractors. This study provides further evidence that movement precision is an important feature of emotional embodiment that should be incorporated in future studies.

## Introduction

It is well-documented that emotional stimuli impact both the cognitive and motor aspects of “goal-directed” behavior (Beatty et al., 2016; Lu et al., 2017). In particular, emotional distracters impact ongoing cognitive processes (Dolcos and McCarthy, 2006). In our daily life, emotional distraction is particularly disruptive to goal-directed behavior. Processing these task-irrelevant emotional stimuli interferes with the performance of a cognitive performance, including working memory and visual searches (Dolcos et al., 2006; Pedale et al., 2019). Distracting emotions might capture attention and reallocate resources used for processing (Ellis and Ashbrook, 1988), consequently impairing cognitive performance. However, how distracting emotions impact motor performance remains unclear. In addition, emotions might influence motor performance differently depending on the type of movement (Jasinska et al., 2012; Gorniak, 2019). For example, when an individual is scared by a spider, it might impact their movement when they were moving a mug from one side of their body to the other. However, this emotional effect would vary depending on the complexity of the moving task. For instance, the emotional effect is typically lower when sliding a mug directly across a desk compared to lifting it above many objects on a desk. Thus, we speculated that the effects of distracting emotions on motor performance could be modulated by the complexity of the movement.

Previous studies examined how emotional stimuli impact motor performance. For example, compared to neutral stimuli, negative emotional stimuli elicit a faster pushing movement (i.e., avoidance), whereas positive images accelerate pulling speed (i.e., approach; Krieglmeyer et al., 2010; Boyd et al., 2011; Önal-Hartmann et al., 2012; Deuter et al., 2014; Hans Phaf et al., 2014). Compared to the speed and the force of movement (Coombes et al., 2006), the precision of movement has received less attention. Emotional stimuli could be either the current target being searched for or task-irrelevant distractors. The impact of “emotional distraction” on goal-directed behavior has been primarily studied in the context of several cognitive behaviors. For example, during the phase of maintaining working memory, the presentation of negative stimuli evokes increased activity in emotional-related areas and decreased activation in working memory-related areas (Dolcos and McCarthy, 2006). Through evaluating stimuli embedded within complex everyday scenes, Pedale et al. (2019) showed that emotional distractors are fixated later, and for a shorter duration, compared to emotional targets, indicating efficient top-down control in avoiding emotional distraction. As such, task-irrelevant emotional distractors might capture attention and impair the performance of on-going motor tasks. Yet, studies investigating how emotional distraction impacts behavior with respect to movement/motion remains limited. Thus, motor performance needs to be evaluated with kinematic measurements to bridge gaps between distracting emotions and motor performance.

The duration of exposure to emotional stimuli impacts the speed and accuracy of movement in different ways (Coombes et al., 2005). In a square-tracing task, the length of exposure to affective stimuli mediates the speed and accuracy of motor performance. Within this task, compared with positive stimuli, negative stimuli led to either increased error (short exposure) or increased speed (multiple exposures). Thus, emotional effects depend the moment in time a given task needs processing resources. Consequently, chronometry measurements could provide valuable insights on the perception of emotion during the execution of movement. As such, it is important to distinguish between and account for the fundamental aspects of movement, which are divided into a premotor phase (from the presentation of a stimulus to the initiation of movement) and an execution phase (from the initiation of movement to the completion of movement; Krakauer et al., 2019). Studies using chronometric measurements and divided response times (reaction time and movement time), previous studies showed that only the reaction time (time from stimulus presentation to initiation of the movement response) is impacted by the presentation of an emotional stimulus (Lu et al., 2017). Compared to the button-pressing task used by Lu et al. (2017), a more complex or more ecological valid motor task is needed to investigate how emotions impact different phases of movement.

Movement complexity is regarded as an important factor influencing the quality of movement. We previously showed that when using “release-move-press” movement (rather than “press only” movement), more complex behavior requires more attention during performance, and that, as behavior becomes more complex, emotional processes impact the quality of movement more (Lu et al., 2017). Thus, different aspects of motor performance are likely impacted by the interaction between the valence of distracting emotions with movement complexity. However, it is not known whether the complex level of movement heterogeneously impacts the quality of motor performance.

Accuracy is usually calculated as the percentage of correct responses when evaluating the quality of cognitive-motor performance. Measurements estimating the quality of movement (e.g., root-mean-squared-error, RMSE) are typically used to assess cognitive-motor tasks (Franks et al., 1982; Coombes et al., 2008; Gentili et al., 2011). Thus, RMSE might provide a rational approach for evaluating the quality of movements, particularly performance precision. Therefore, the current study combined measurements of accuracy and RMSE in a cognitive-motor task to explore the elemental aspects of human motor performance.

This study investigated the interaction between distracting emotional stimuli and movement complexity on the quality of motor performance. A modified oddball paradigm was utilized to separate the emotional stimuli as distractor, but sharing the same response process (Yuan et al., 2007, 2017; Lu et al., 2017). Response time and movement time were measured to assess performance speed, while accuracy and RMSE were determined by evaluating the precision of the motor performance in a cognitive-motor task. Regarding chronometric measurements, we hypothesized that cognitive-motor performance would be differentially affected by the distracting emotional stimuli. Specifically, we predicted that the reaction time would be extended by the distracting emotional stimuli compared to the neutral situation. In comparison, movement time was expected to take longer when performing more complex movement. We also hypothesized that there would be an interaction between the distracting emotional stimulus and movement complexity on accuracy, with the highest precision in motor performance occurring in the presence of a positive stimulus under more complex movement.

## Methods

### Participants

Forty-two students from Shanghai University of Sport participated in this study. The ages of the participants ranged between 18 and 28 years old (mean = 21.1 years old, standard deviation = 2.24 years old). All participants had a normal or corrected-to-normal vision. No participants suffered from an emotional disorder or were taking medications that affect the nervous system. The G^*^power (Faul et al., 2009; V3.1.9.7) was used to determine the sample size. The calculation focused on the current hypothesis, that the comparison between movement performance under positive or negative background. In the current design, the sample size was required for six repeated measurements with the expected effect size of 0.18 (Lu et al., 2017) and a significance level of 0.05 at the desired power of 0.85.

The local ethics committee approved this study, and all participants had signed the informed consent before initiation of the experiment. All participants were right-handed as determined by the Edinburgh Handness Inventory (mean = 77, standard deviation = 24). Each participant was paid 50 RMB after finishing the experiment.

### Stimuli

**Figure 1:**
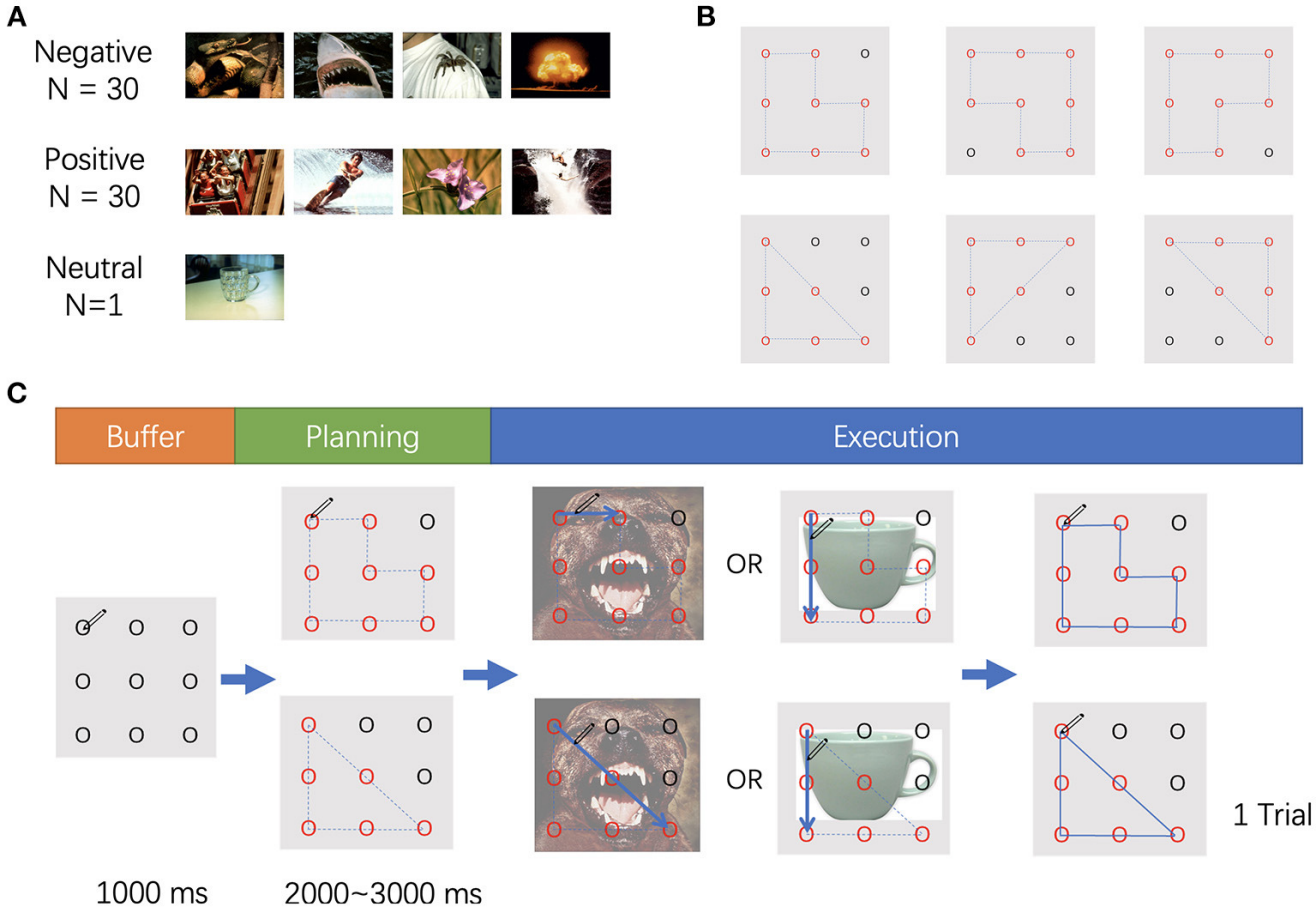
**(A)** Samples of stimuli used in the experiment. **(B)** The movements used in the experiment. **(C)** The procedure of the experiment.

The stimuli employed in this study included affective images selected from the International Affective Pictures System^1^ (Figure 1A) (Lang et al., 1997) and two different geometric shapes (i.e., triangle and polygon) (Figure 1B). The affective images were selected based on normative rating of valance and arousal, also avoiding the human face or erotic pictures. A total of 60 pictures were utilized and categorized into two types of stimuli: positive or negative, according to the valence value. In addition, one picture with a mug on a table was selected as a neutral stimulus (valence: 4.98, arousal: 2.66). All pictures were adjusted to a size of 15 cm × 10 cm, 100 pixels per inch. Two *t*-tests were conducted for the valence and arousal, respectively. The valence of the positive stimuli was significantly higher than that of the negative stimuli (*p* < 0.001), and there was no difference in arousal between the two types of stimuli (*p* = 0.745). The valence and arousal values are displayed in Table 1.

**Table 1:** The mean ± standard deviation values for valence and arousal in each condition.

	**Positive**	**Negative**	***t*-value**	***p*-value**
Valence	7.33 ± 0.35	3.55 ± 0.53	31.75	<0.001
Arousal	4.93 ± 1.51	5.05 ± 1.40	0.33	0.745

*The difference between these two shapes is the number of turning points. The polygon contains five turning points, while the triangle contains only two. We considered drawing the polygon was more complicated than drawing the triangle*.

### Procedure and Apparatus

After signing the informed consent, each participant was asked to complete the State-Trait Anxiety Inventory to assess their anxiety state. Only those who were within the normal range (<50) on the assessments proceeded to the experiment. The participants were asked to sit ~70 cm away from the screen and the resolution of the screen (ST2420L, Dell) is 1920 by 1080. The stimuli were presented on the screen with horizontal and vertical visual angles <6°, and a digital tablet (Intuos Pro L, Wacom) was placed on the desk in front of each participant. The experiment consisted of one practice block (10 trials) and four blocks in the formal experiment. Each block contained 100 trials. Stimulus display and behavioral data acquisition were performed using MATLAB 2019a (Mathworks, Natick, MA, USA).

In the task, nine points were displayed on the screen, separated by the same distance. The points were presented on the screen with horizontal and vertical visual angles <10°. The participants were asked to hold a pen, place it on the upper left point on the digital tablet, and wait to start. The nine points were presented for 1,000 ms, then some of the outlines of the points turned into red and blue dotted lines connected these red points to form a triangle or a polygon. The participants were asked to recognize the shape according to the red points and blue dotted lines, which appeared randomly between 2,000 and 3,000 ms after initiation of the experiment. Then the affective picture appeared as the background, and the dotted lines disappeared at the same time. The participants were instructed to draw the trajectory once the picture appeared. If the picture was a mug (neutral), the direction of the drawing should be counterclockwise; if another image was shown, the direction of the drawing should be clockwise. When the participants completed drawing and the pen was returned to the upper left point, the trial ended (Figure 1C).

To avoid contamination across emotions, the block design was used in the current study. Only a single type of affective stimulus was presented in each block. Each block consisted of 70 neutral stimuli and 30 affective stimuli (30 positive or 30 negative), and a total of 100 trials was presented randomly. Two blocks of positive stimuli followed by two blocks of negative stimuli, or the opposite order, were presented. The block sequence was counterbalanced across the participants.

Before the formal experiment, the participants had two practice sessions. The first one was to familiarize the participants with the operation of the tablet. They were told to draw whatever they wanted on the tablet with Paint (Microsoft, Redmond, WA, USA). The second practice session contained 10 trials to familiarize the participants with the procedure. The neutral stimulus was the same as that in the formal experiment, whereas the affective stimuli for the practice session were not used in the formal experiment. Three-minute breaks were allowed between blocks.

### Data Analysis

A tablet equipped with MATLAB software was employed to collect and analyze the kinematic characteristics of the task performance. The sampling frequency for the pen sensor was set at 200 Hz, and all locations were calibrated to the upper left circle, which was set as the beginning and ending point.

The reaction time, movement time, accuracy, and RMSE were computed to quantify the cognitive-motor performance. The reaction time was measured as the duration from the onset of the stimulus to the moment that the pen left the upper left point. The movement time was measured as the duration from the pen leaving the upper left point until it returned back to the upper left point, then it was calibrated to the shape length. In computing the accuracy, the trials with tracing in the wrong direction or missing any points in the trajectory were regarded as incorrect. The RMSE was calculated with the correct trials only with the following formula:

$$\begin{array}{l} RMSE=\sqrt{\frac{\sum_{i=1}^{N}\left[{\left(x_{a}-x_{i}\right)}^{2}+{\left(y_{a}-y_{i}\right)}^{2}\right]}{N}} \end{array}$$

### Statistical Analysis

Analysis of variance (ANOVA) was used to separately assess the accuracy, RMSE, reaction time, and movement time of the three emotional stimuli (neutral, positive, and negative) and the two movement types (drawing the polygon, DP and drawing the triangle, DT). Furthermore, considering the standard stimuli (neutral condition) kept the same picture in the oddball paradigm, we conducted a two-way ANOVA within the neutral as the baseline for RMSE, reaction time, and movement time. The significant result was reported. Greenhouse–Geisser corrections were applied when sphericity was violated, and the effect sizes were calculated using $\eta_{p}^{2}$. All *post-hoc* pairwise comparisons were adjusted using Bonferroni corrections to counteract the problem of multiple comparisons. The alpha significance level was set at 0.05.

## Results

### Accuracy

The interaction between the movement type and the emotional stimulus was significant [*F*_(2, 82)_ = 12.953, *p* < 0.001, $\eta_{p}^{2}$ = 0.240]. The *post-hoc* tests showed that there was no difference in the accuracy among the three emotional stimuli for the DP; while for the DT, the accuracy with a negative stimulus (0.783 ± 0.139) was significantly less than with a positive (0.833 ± 0.087, *p* = 0.012) or neutral stimulus (0.881 ± 0.084, *p* < 0.001). In addition, the difference in the accuracy between the neutral and positive stimuli was also significant (*p* < 0.001). On the other hand, there was no significant difference in the accuracy between DP and DT with a neutral or positive stimulus, while the difference was significant with a negative stimulus (*p* < 0.001).

**Figure 2:**
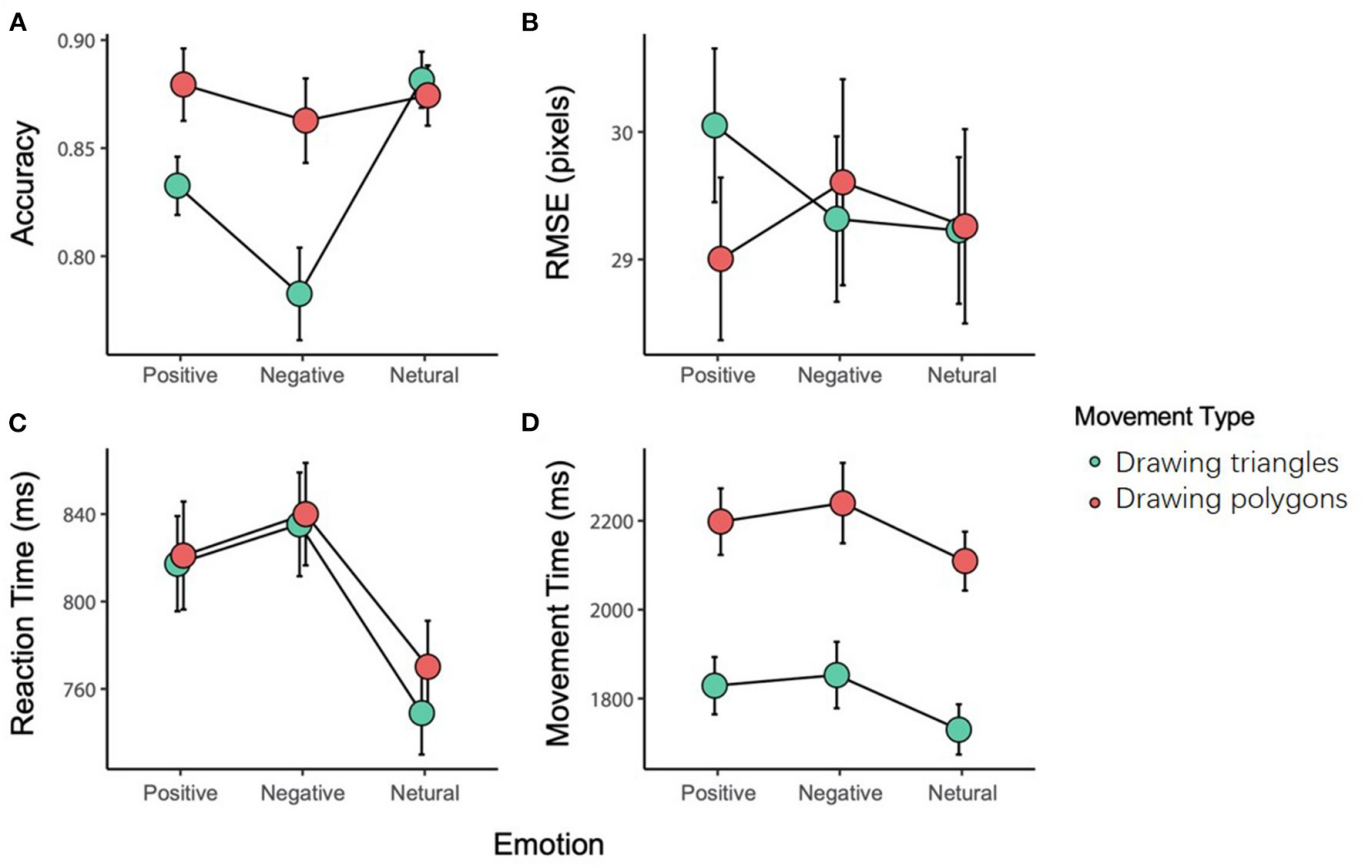
The results of the variables [(**A)** accuracy, **(B)** RMSE, **(C)** reaction time, **(D)** movement time].

The main effect of emotional stimuli was significant [*F*_(2, 82)_ = 13.094, *p* < 0.001, $\eta_{p}^{2}=$0.242], and further analysis revealed that the accuracy with a negative stimulus was significantly less than that with a positive (*p* = 0.030) or neutral (*p* < 0.001) stimulus. Also, the main effect of the movement type was significant [*F*_(1, 41)_ = 9.783, *p* < 0.001, $\eta_{p}^{2}\;=$ 0.193], with DP showing a higher accuracy compared with DT (Figure 2A).

### RMSE

The interaction between the movement type and the emotional stimulus was significant [*F*_(2, 82)_ = 3.941, *p* = 0.023, $\eta_{p}^{2}$ = 0.088]. The *post-hoc* tests showed that with a positive stimulus, DP (30.052 ± 0.603) induced a greater RMSE than the DT (29.005 ± 0.603, *p* = 0.024). There was no significant difference in the RMSE of the movement with a negative or neutral stimulus (Figure 2B).

After baseline correction, the interaction was kept [*F*_(1, 41)_ = 5.281, *p* = 0.027, $\eta_{p}^{2}$ = 0.114]. The *post-hoc* tests showed the similar effect in RMSE.

### Reaction Time

Two-way repeated-measures ANOVA of the reaction time showed a significant main effect of the emotional stimuli [*F*_(2, 82)_ = 13.615 *p* < 0.001, $\eta_{p}^{2}\;=$ 0.249]. Further analysis showed that the reaction time with a neutral stimulus (0.761 ± 0.020 ms) was shorter than that with a negative (0.837 ± 0.024 ms, *p* < 0.001) or positive stimulus (0.822 ± 0.023 ms, *p* < 0.001), while no difference was found between the negative and positive stimuli (*p* = 1.000). There was no other main effect or interaction (Figure 2C).

After baseline correction, we didn't find any significant results for the main effect or interaction.

### Movement Time

The main effect of the movement type was significant [*F*_(1, 41)_ = 339.241, *p* < 0.001, $\eta_{p}^{2}$ = 0.892]. Further analysis showed that the adjusted movement time for DT (1.804 ± 0.426 ms) was significantly shorter than that for DP (2.182 ± 0.503 ms, *p* < 0.001). There was no other main effect or interaction (Figure 2D).

After baseline correction, we didn't find any significant results for the main effect or interaction.

### Comparison of the Presentation Sequence

Considering the potential influence of picture presentation order, a control analysis was conducted with a three-way ANOVA. The presentation (first and second), emotional stimuli (positive and negative), and the movement types (drawing the polygon, DP and drawing the triangle, DT).

The main effect of presentation was found for the movement time [*F*_(1, 41)_ = 122.896, *p* < 0.001, $\eta_{p}^{2}$ = 0.750], and reaction time [*F*_(1, 41)_ = 17.366, *p* < 0.001, $\eta_{p}^{2}=$ 0.298], showing the longer movement time and reaction time with the first picture presentation, comparing to the second presentation. However, we didn't find any interaction on the presentation.

## Discussion

This study investigated the impact of distracting emotional stimuli on the cognitive-motor performance of different types of movement. Targeted movement was impacted by distracting emotional stimuli in terms of both speed and the precision of drawing.

We showed that the distractor stimuli (i.e., emotional stimuli) induced a longer reaction time compared to standard stimuli (i.e., neutral stimuli). Adjusted movement time was longer when drawing the polygons compared to triangles. Negative stimuli were only associated with reduced accuracy compared to positive and neutral stimuli when participants performed less complicated movement (i.e., drawing the triangle). In contrast, for distracting positive stimuli, RMSE was greater for those drawing polygons compared to triangles. Our findings support those of previous studies using the key-pressing task in the presence of emotional stimuli, along with the concept that movement precision (as assessed by the RMSE) is moderated by both the movement type and distracting emotions (Lu et al., 2017).

Our results showing the effect of distracting emotional stimuli on accuracy support those of previous studies (Fanselow, 1994). Specifically, movement accuracy was lower in the presence of negative and positive distractors compared to standard ones. Furthermore, these effects had different patterns when drawing complicated versus less complicated shapes. Compared to positive stimuli, only the negative distractor impacted the accuracy of movement for complicated drawings. This might be explained by the fact that negative distractors potentially contain dangerous or threatening information, driving different allocations to attention (Soares et al., 2009). The more complicated task had higher accuracy, which might be because it required more focused attention compared to the less complicated task (Murphy et al., 2016). In contrast, previous studies obtained different outcomes when comparing the effects of negative and positive stimuli. Some studies showed that negative stimuli tend to impair accuracy more than positive stimuli (Coombes et al., 2005), while others obtained the opposite results (Most et al., 2007). Our study implied that this emotional effect could be modulated by the complexity of the motor task. A positive background did not influence the accuracy of completing the drawing task. Interestingly, accuracy was only impaired by a negative distractor when the task was relatively less complex. During motor planning, more complex movement (i.e., drawing polygons) required more attention at the start of movement.

To investigate the accuracy in a refined way, we used RMSE to assess the precision of the movement. As expected, the RMSE values showed a different trend to accuracy values. The RMSE values of different movement types varied in the presence of positive backgrounds but not with negative backgrounds. RMSE measures the quality of movement performance by describing the precision of drawing performance. Interestingly, after the positive background appeared, polygons were drawn with greater precision compared to triangles. Usually, polygons are considered to be more complex to draw than triangles. We also expected that the participants would perform better (i.e., more precision) in drawing less complicated shapes. However, this phenomenon was only detected with the negative distractor, although the results were not significant. After exposure to the positive distractor, the more complicated movement (i.e., drawing polygons) might have required more attention compared to the less complicated movement (i.e., drawing triangles), resulting in greater precision (i.e., higher RMSE) for the complicated movement. However, the effect of emotion on precision has not been previously reported. We showed that RMSE varied, allowing minor errors that are ignored by accuracy indices to be captured. Thus, RMSE is an effective measure of kinematic indices.

The reaction time and movement time after exposure to emotional stimuli were generally supported previous studies. The only difference was that the reaction time was longer after emotional distractors (both positive and negative) compared to neutral stimuli. This result might be attributed to the experimental design, rather than the properties of the pictures. Shorter reaction times in the current study were invoked by the standard stimulus, which appeared more times than the deviated stimuli (i.e., emotional stimuli). Consequently, the less frequent deviated stimuli would have longer reaction times compared to the standard stimuli, as previously detected by our group. When using a modified oddball task, the reaction time in the presence of deviant stimuli are processed *via* the same cognitive process; consequently, the reaction times to these deviant stimuli tend to be similar (Yuan et al., 2007; Naugle et al., 2010, 2011; Lu et al., 2017). A previous study also reported that the movement time was impaired when participants were briefly exposed to negative stimuli compared to positive stimuli (Coombes et al., 2005). However, neither reaction time nor movement time was significantly different after the appearance of positive stimuli compared to negative stimuli in the current study. This discrepancy might be caused by arousal. In general, arousal tends to be greater in response to negative stimuli compared to positive stimuli (Lang et al., 1993; Feng et al., 2014; Lu et al., 2017). Besides, our control analysis showed, comparing to the first presentation, the reaction time and movement time was shorter with the second presentation. The emotional adaption might reduce the influence on the movement performance. However, further study should be taken to investigate this speculation.

The current study aimed to characterize the influence of emotional stimuli on different types of motor performance. The modified oddball paradigm was used to provide the same cognitive processing, and to compare the effects of distracting emotional stimuli. We demonstrated that both valence and movement types interacted to affect accuracy and RMSE. Accuracy increased when participants were exposed to negative distractors during less complicated movement. In comparison, the performance precision of complicated movement was greater in more complicated movement compared to less complicated movement, especially in the presence of positive distractors.

Although the paradigm that we utilized in the current study was verified in many previous studies, this paradigm was limited by neutral stimuli having a weakened function. The ratio of stimuli could be modified in future studies. Also, working memory processes were involved in the current experimental design, but the influence was not measured. Further study should consider the interplay between emotions and working memory (Buttafuoco et al., 2018). Last but not least, an electroencephalogram or near-infrared spectrum could be used in future studies to evaluate the brain activities on the examined phenomena (Chen et al., 2020).

Overall, both the type of emotion and type of movement were associated with the quality of movement performance. Movement time was primarily affected by movement complexity. In the presence of negative emotional distractors, movement complexity impacted accuracy. In contrast, in the presence of positive emotional distractors, movement type affected precision. In conclusion, movement precision is an important feature of emotional embodiment that should be incorporated in future studies.

## Data Availability Statement

The raw data supporting the conclusions of this article will be made available by the authors, without undue reservation.

## Ethics Statement

The studies involving human participants were reviewed and approved by the ethics committee of the Shanghai University of Sport. The patients/participants provided their written informed consent to participate in this study.

## Author Contributions

YL: designed the experiment and wrote the manuscript. TW: collected the data, analyzed the data, and wrote the manuscript. QL: collected the data. ZC: wrote the experiment codes. All authors contributed to the article and approved the submitted version.

## Conflict of Interest

The authors declare that the research was conducted in the absence of any commercial or financial relationships that could be construed as a potential conflict of interest.

## Footnotes

^1^The IAPS stimulus numbers are as follows: Negative-1040, 1050, 1052, 1113, 1120, 1200, 1201, 1930, 2722, 2752, 3250, 5120, 5971, 6210, 6834, 7060, 7234, 8480, 9000, 9010, 9046, 9090, 9101, 9110, 9220, 9280, 9390, 9390, 9622, 9630; Positive- 1600, 1604, 1610, 1620, 2360, 5000, 5001, 5010, 5200, 5201, 5220, 1603, 5780, 5891, 7545, 8161, 5470, 5621, 5626, 5629, 8034, 8080, 8170, 8180, 8190, 8200, 8370, 8470, 8490, 8501; Neurtal-7035.
